# Psychosis Associated with Acquired Porencephaly—Cause or Incidental Finding? Case Report and Review of Literature

**DOI:** 10.3390/medicina58050586

**Published:** 2022-04-24

**Authors:** Maria Gabriela Puiu, Vlad Dionisie, Alexandru Cristian Filip, Mirela Manea

**Affiliations:** 1 Department of Psychiatry and Psychology, ‘Carol Davila’ University of Medicine and Pharmacy, 020021 Bucharest, Romania; mg_puiu@yahoo.com (M.G.P.); mirelamanea2003@yahoo.com (M.M.); 2 Department of Psychiatry, ‘Prof. Dr. Alexandru Obregia’ Clinical Hospital of Psychiatry, 041914 Bucharest, Romania; 3 Department of Radiology and Medical Imaging, ‘Dr. Carol Davila’ Central Military Emergency University Hospital, 010825 Bucharest, Romania; alexandru.filip95@yahoo.com

**Keywords:** porencephaly, psychosis, frontal lobe, organic brain disorder, cyst, encephalomalacia, organic personality

## Abstract

Porencephaly, a rare disease affecting the central nervous system, is represented by a cerebrospinal fluid-filled cavity in the brain. There are two types of porencephalic cavities: congenital and acquired. Porencephaly is mainly associated with neurological and developmental consequences. Associated psychotic symptoms were reported in a few cases, and due to this fact, there is a knowledge gap regarding the diagnostic and therapeutic approach to such cases. We present the case of a 32-year-old male diagnosed with a psychotic disorder associated with acquired porencephaly. The porencephalic cystic lesions were most probably due to a traumatic brain injury at the age of 6 years old. The psychotic symptomatology consisted of interoceptive/visceral hallucinations, delusions with persecutory and religious/magic content and disorganised behaviour. The porencephalic cavity was confirmed by a computed tomography scan. The patient was treated over the course of time with risperidone, olanzapine and zuclopenthixol. The existing literature regarding other cases of psychosis associated with porencephaly is discussed. In conclusion, even though porencephaly was asymptomatic for a long period of time, we argue that there is a causal relationship between the chronic psychotic symptoms and the porencephalic cyst in our case.

## 1. Introduction

Porencephaly is a rare disease of the central nervous system characterised by the existence in the brain of degenerative cavities filled with cerebrospinal fluid [1]. According to many authors, there are two types of porencephaly: true porencephaly, also named congenital porencephaly, and acquired porencephaly, also named pseudo porencephaly [2,3,4]. Congenital porencephaly includes brain lesions described as being the consequence of developmental abnormalities of cell migration often associated with other malformations of the brain. Acquired porencephaly includes brain lesions that occur as a consequence of any destructive condition caused by vascular, infectious or traumatic incidents. Familial cases of porencephaly are thought to be caused by mutations of the COLI4A1 gene that leads to brain small-vessel disease with haemorrhage [5]. Clinical manifestations of porencephaly vary greatly as the lesions differ in size and location. Hemiparesis and epilepsy are the most common clinical manifestation [2]. Cognitive deficits may range from a mild learning disability to severe mental retardation [6].

Few cases of psychosis associated with porencephaly are reported in the scientific literature. For the discussion part, we also conducted a literature review of other cases of porencephaly associated with psychotic symptoms. For this purpose, we performed a search in the PubMed database using the following terms: ‘psychosis’ or ‘schizophrenia’ cross-referenced with ‘porencephaly’. We screened the selected articles’ references for relevant studies that could be included in this paper. Only articles in English were selected.

In this article, our aim is to present a case of a patient with acquired porencephaly associated with psychosis and the therapeutic challenges encountered during care. In addition to presenting the course of our case, we propose to review the similar cases found in the literature and provide up to date data to clinicians facing similar patients and decision-making challenges.

## 2. Case Presentation

A 32-year-old male patient was admitted to ‘Prof. Dr. Alexandru Obregia’ Clinical Hospital of Psychiatry, in Bucharest, for psychotic symptomatology. He was willingly brought into the psychiatric emergency unit by ambulance and police. During the last two days prior to hospital admission, the patient started to be frightened because he thought that different people were laying spells on him. He reported feeling the spells entering his body as well as seeing ghosts. For these reasons, the patient called the ambulance and the police to help him. During psychiatric evaluation at the emergency room, the patient said that the spells came out of him during transportation. He was sure that a device from the ambulance took the spells away.

The mental state examination revealed a patient oriented in person, place and time with a suspicious rapport. The patient’s thought content was characterised by persecutory and religious/magic delusions (being followed by persons that were laying spells on him). The patient did not admit to experiencing any auditory hallucinations but admitted to interoceptive/visceral hallucinations (he felt the spells throughout his entire body). At admission, the patient was very anxious and with behaviour characterised by agitation and disorganisation. During hospitalisation, he was observed muttering to himself. His speech was often circumstantial, and he presented concrete thinking. The patient had an irritable mood, dysphoric and anger outbursts, low impulse control and frustration tolerance. He denied current suicidal thoughts. The Modified overt aggression scale (MOAS) score (on admission): verbal aggression-3 points (impulsively threatens violence toward others or self). The Positive and Negative Symptoms Scale (PANSS) score (on the second day of admission) was: total—89, positive symptoms—26, negative symptoms—17, general psychopathology—46; Raven’s progressive matrices: 67; frontal assessment battery score: 9/18 (important impairment of frontal executive function); Mini-Mental State Examination (MMSE): 30/30 points. Due to his symptomatology and refusal to be admitted, he was hospitalised under compulsory admission.

The patient denied any personal or family psychiatric history. He was living alone in a rented flat in an urban area, was never married and had no children. He completed basic upper school (ten grades) and had been working until admission as a qualified worker. He denied using tobacco, alcohol or other psychoactive substances. The collateral history provided by the patient’s mother and sister revealed no peripartum pathologies but a traumatic brain injury (TBI) at the age of 6 followed by loss of consciousness and hospitalisation. The patient did not undergo any neurosurgery. According to his mother, he recovered well except for the episodic amnesia of the event in question. The information retrospectively attained during the interview showed that as a child he was playful, easy to get along with and sociable without any conduct disorders. Indeed, his mother noticed over the years that the patient was more impulsive and easily irritable after the TBI, but this did not interfere with the educational or social domains nor required a specialist assessment. The patient attended mainstream schools and received general education. He had a below average educational path, but he managed to graduate ten grades. In his mother’s opinion, he had, in general, a normal development as a child and adolescent, without any noteworthy physical or emotional traumatic events, except for the TBI (he had friends, and he was involved in group social activities). Moreover, he had a normal life course. After finishing ten grades, he completed training and apprenticeship for a vocational qualification, and then he successfully pursued a career on that path. She did not notice any change while talking to him on the phone in the days prior to his hospital admission or when he visited her two weeks before.

**Figure 1:**
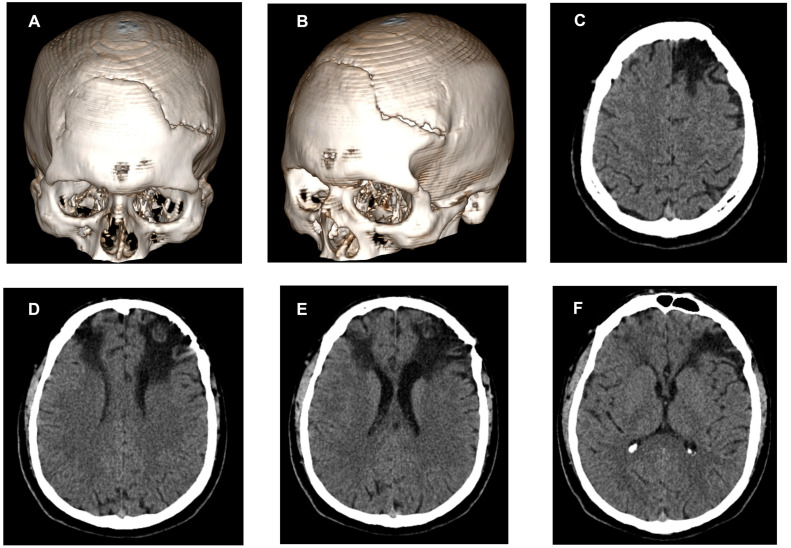
Computed tomography 3D bone reconstruction (**A**,**B**) and axial computed tomography of the brain (**C**–**F**). A post-traumatic bone defect of the skull can be seen in (**A**–**F**) shows changes of encephalomalacia in the frontal lobes’ parenchyma, affecting both white and grey matter, more extended on the left side, with enlargement of the anterior ventricular horn (aspect of acquired porencephalic lesions).

The physical examination revealed no pathological signs or symptoms. We performed haematological, biochemical and toxicological tests, which were in the normal range, thus excluding a potential medical condition. Considering that this was the patient’s first episode of psychosis, a computed tomography (CT) scan was performed (Figure 1).

The CT showed a post-traumatic bone defect seen in the frontal area of the skull, more extended on the left side, with non-union of the bone segments (Figure 1A,B). Furthermore, the result revealed extended hypodense areas of focal parenchymal atrophy in both frontal lobes with replacement by a cystic/parafluid mass (densities between 0 and 15 Hounsfield units) that extends from the enlarged pericerebral subarachnoid frontal space to the anterior ventricular horns (acquired porencephalic lesions). These changes of encephalomalacia in the parenchymal brain tissue are more extended on the left frontal lobe, affecting cortical grey matter and subcortical areas of superficial and deep white matter located anteriorly in the superior, middle and inferior frontal gyri, corona radiata and centrum semiovale. On the right frontal lobe, the aspect is similar but less extended, affecting cortical and subcortical areas of superficial and deep white matter located anteriorly in the superior and middle frontal gyri. The ventricular system is slightly asymmetric with dilatation of the left anterior ventricular horn and traction to the porencephalic changes in the frontal lobe (Figure 1C–F). One of the differential diagnoses of porencephalic lesions can be open lip schizencephaly, but in our patient, the lesions are not lined with grey matter.

After receiving the results of the CT scan, the patient was again asked if he had any previous CT scan or MRI examinations or any other types of imaging investigation of his brain, but he denied. He also denied having any head injury throughout his life.

The neurological examination was normal, with no focal deficits or other abnormalities.

The patient was prescribed a second-generation antipsychotic (i.e., risperidone) and a benzodiazepine (i.e., diazepam 20 mg/day) in order to reduce the anxiety level and improve his sleep. The antipsychotic was gradually increased up to 6 mg/day during the inpatient stay with no appearance of side effects and the diazepam was discontinued until discharge.

Considering the clinical presentation, neuroimaging data and frontal lobe dysfunction, we argued that there is a relationship between the occurrence of psychotic symptoms and porencephaly. For this reason, the diagnosis at discharge was ‘psychotic disorder due to a general medical condition’ and ‘organic personality disorder’.

One year later, the patient had a second inpatient admission under similar circumstances. On the mental state examination, the patient was oriented in person, place and time, was highly suspicious and hypervigilant and reported visual hallucinations of a man he knew was paralysed walking behind him and hiding to follow him. Furthermore, the patient had persecutory delusions (his relatives wanting to harm him). He was verbally and physically aggressive and had clastic behaviour and abrupt changes in attitude. Since his first psychiatric admission, the patient reported he had been compliant with the psychiatric treatment. He changed his job twice and also moved to a different house (he was living with some relatives). The second resignation happened just a few weeks before admission and he was looking for a new job during this time. This could be a potential stressful event that could contribute to the psychotic relapse. His mother stated that the patient’s behaviour and attitude changed in the last year as he became repeatedly verbally aggressive towards her and changed jobs and living places due to not getting along with his work colleagues and his neighbours, respectively. The Modified overt aggression scale score (on admission): 16 (4 points—verbal aggression, 2 points—aggression against property, 2 points—physical aggression). The PANSS score (second day of hospital stay) was: total—101, positive symptoms—33, negative symptoms—18, general psychopathology—50; MMSE score was: 30/30 points.

Initially, the patient received risperidone (6 mg/day) for a short period of time, and then, due to inadequate clinical response, the antipsychotic drug was switched to olanzapine (up to 20 mg/day). As there was no evidence of improvement on olanzapine, the aggressive behaviour persisted and there were doubts regarding his future compliance to oral medication as well, he was started on zuclopenthixol acetate 50 mg/mL, 1 mL every 3 days and then to zuclopenthixol decanoate 200 mg/mL 1 ml every 2 weeks. The long acting formulation treatment was preferred by the patient. He was discharged after 16 days with the diagnosis of ‘psychotic disorder due to a general medical condition’ and ‘organic personality disorder’. At the 4-week follow-up, the patient was continuing his treatment and started a new job.

## 3. Discussion

Herein, we report a novel case of a patient with psychotic symptoms and acquired porencephaly diagnosed during hospitalisation for his first episode of psychosis. The case is documented by a thorough clinical assessment and anamnesis and paraclinical investigations, including a CT scan. The case highlights a male patient with no psychiatric history who presented with psychotic symptomatology. The patient had no neurological signs and was initially treated successfully with risperidone. Over the course of his illness, the patient failed to respond to risperidone or olanzapine, but his psychotic symptoms were managed successfully with zuclopenthixol. 

The existing literature on this subject comprises only five case reports of patients with psychotic symptoms and porencephaly (Table 1). Three of these cases concerned female patients, two of them being younger than our patient (25 and 26 years old, respectively) [7,8,9]. Only Uvais et al. (2020) and Boyer et al. (2011) reported a case of a male patient with an acute first episode of schizophrenia associated with Capgras syndrome and a case of a male with schizophrenia and porencephaly due to most probably encephalitis, respectively [10,11].

An interesting feature of our case is the absence of any neurological deficit, which is similar to some of the other previous cases [8,9,10]. Porencephaly is usually associated with cerebral palsy, hemiparesis, epilepsy, mental retardation or other learning disabilities and hemiplegia [2,12].

In four of the reviewed cases, the cysts were located in the frontal lobes, while in three patients, the temporal lobe was an additional site [7,8,9,10,11]. In the case presented herein, the porencephalic cavity affected the frontal lobes. This localisation of porencephalic lesions is compatible with the occurrence of psychotic symptoms. Although a generalised brain involvement has more compelling evidence, the scientific literature documents specific structural brain abnormalities involving the frontal and temporal lobes in patients with schizophrenia or first episode of psychosis [13,14,15]. Furthermore, qualitative brain imaging evaluations in patients with psychosis outlined the importance of neurodevelopmental anomalies, such as porencephalic cysts, and their possible causal role [15]. This may offer some explanations on how the frontal lobe defects may contribute to either increased vulnerability, accelerated onset of disease or development of certain psychotic-spectrum symptoms. Tylš et al. (2019) argued that even diffuse microscopic changes in the neural networks might result in a schizophrenia-like clinical picture no matter the structural findings on computed tomography or magnetic resonance imaging [16]. Our case displays a distinct characteristic in this regard since the probable cause of porencephaly was established to be the lesions due to an important TBI in childhood rather than a congenital one. Moreover, the patient’s condition can be viewed as a component of the current neurodevelopmental approach model of psychosis [17].

In most of the previous cases documented in the literature, the antipsychotic of choice was a second-generation one, namely olanzapine, in a dose of 10 mg/day, with a good clinical response. Usually, first-episode patients respond promptly to antipsychotic treatment. Williams et al. (2016) suggested that treatment-resistant symptoms may appear also in the context of brain malformations or developmental disorders [18]. The case documented here shows several challenging aspects. First of all, despite an apparently good compliance to the antipsychotic treatment, the patient had difficulties in impulse control and proneness to threats and violence. These could appear in the context of a change in the development of personality as a consequence of TBI. Secondly, the treatment varied due to poor response. More precisely, the antipsychotic choices ranged from risperidone to olanzapine, and in the end, the patient’s symptomatology was managed successfully with zuclopenthixol. Quarantelli et al. (2014) showed that antipsychotic non-responder patients had a more severe frontal atrophy [19]. Taking into consideration that our patient’s defects were located in the frontal lobe, we could hypothesise that this is one of the possible explanations for the failure of two antipsychotic trials. Furthermore, clinicians should keep in mind that such difficult to treat cases could reveal, in an in-depth view, a more complex pathology.

Regarding the clinical picture of our case, besides common delusions, the patient experienced visual hallucinations, which are atypical and less common than auditory hallucinations in people with psychotic disorders such as schizophrenia [20]. Furthermore, visual hallucinations are often regarded as a marker for a possible organic aetiology such as epilepsy, migraine or Parkinson’s disease [21]. Noyan et al. (2016), Douzenis et al. (2010) and Uvais et al. (2020) reported no hallucination of any type in their cases [7,9,10]. This is the first reported case in which the patient presents both auditory and visual hallucinations. Corroborated with the heteroanamnesis, which pointed to brain trauma, we decided to perform a neuroimaging investigation. Taking all of this into consideration, in such cases, some particular symptoms or personal data should be looked at carefully or be questioned, as they may lead to imaging investigations of most importance for a complete evaluation.

Another novelty aspect of this case in comparison with other cases described in the literature is that the patient also had symptoms of organic personality change such as anger outbursts, impulsivity, frustration and aggression. In addition, the frontal assessment battery showed an important frontal lobe dysfunction in our patient. Other cases of porencephaly associated with psychotic symptoms found in the literature did not mention symptoms of associated personality disorder or did not specifically assess frontal lobe functions. Damages to the frontal lobe are particularly implicated in personality disorders due to consequences of TBI [22].

Since even just a TBI can play an important part in the development of psychosis at any point in the patient’s life, cases of porencephaly are also at a higher risk of developing a psychiatric disorder compared to the rest of the population. Psychiatric and neurological follow-up at different time-points of children with a history of TBI could help in the early detection of porencephaly. Secondary preventive interventions already developed for primary psychosis, such as schizophrenia, should be employed soon afterwards. Of these, school-based mental health support programmes, interventions for stress and anxiety management and identification of subjects during the prodromal stage could be implemented in order to prevent transition to psychosis [23].

## 4. Conclusions

In conclusion, our case report of a patient with acquired porencephaly and associated psychosis enriches the current scarce knowledge on this subject and reinforces the importance of brain imaging and detailed anamnesis in similar cases of psychosis. Moreover, this case supports the theory that certain neurological brain defects can be asymptomatic, and therefore, patients are easily overlooked until it later manifests as a certain psychiatric disorder. As there is a limited number of cases of porencephaly associated with psychosis described in the literature, we consider that any case report and review will contribute important information for further approach and management of such cases.

## Figures and Tables

**Table 1:** Case reports of porencephaly associated with psychosis in the literature.

Case Report	Patient’s Gender, Age, Diagnosis	Personal and Family History, Neurodevelopmental Issues	Neurological Evaluation	Psychological/Psychiatric Standardised Investigations	Neuroimaging Findings	Treatment
Dounezis et al., 2010 [7]	female, 25 yo, first psychotic episode	Porencephaly in the front-temporal lobes region since birth; mild mental retardation; spastic paresis in left arm	Weakness and slight spastic paresis in right arm	Initially: PANSS: 74 MMSE: 30/30 WAIS: 64 After treatment: PANSS: 47	MRI: large porencephalic cyst on the left frontal and temporal lobes; atrophy in the left side of brainstem due to Wallerian degeneration.	Olanzapine 10 mg/day
Boyer et al., 2011 [11]	male, 46 yo, paranoid schizophrenia	Encephalitis at the age of 6 mo with mild spastic right hemiparesis sequelae	mild spastic right hemiparesis	PANSS: +25, −17, general psychopathology 32 MMSE: 30/30 WAIS: 89, verbal 91, performance 93	MRI: porencephalic cyst in the left frontal and temporal lobes	Risperidone 4 mg/day Cyamemazine 25 mg/day
Hussain et al., 2015 [8]	female, 26 yo, first psychotic episode	None	NAD	MMSE: 30/30 IQ: normal range (value not provided)	MRI: large porencephalic cyst on the left side of FL	Olanzapine 10 mg/day
Noyan et al., 2016 [9]	female, 43 yo, first psychotic episode	None	NAD	N/D	MRI: extensive porencephalic cyst in the right medial FL with mild mass effect on the parenchyma	Olanzapine 10 mg/day
Uvais et al., 2020 [10]	male, 30 yo, first episode of schizophrenia	N/D	NAD	N/D	MRI: porencephaly in the right OT region with agenesis of splenium of the corpus callosum	Olanzapine 10 mg/day
Our case	male, 32 yo, first and second psychotic episode	TBI (at the age of 6 yo)	NAD	1st episode: PANSS: 89 MMSE: 30/30 MOAS: 3 FAB: 9/18 2nd episode: PANSS: 101 MMSE: 30/30 MOAS: 16	CT: encephalomalacia changes in the frontal lobes with acquired porencephalic cystic lesions, more extended on the left side and with enlargement of the anterior ventricular horn	1st episode: risperidone 6 mg qd 2nd episode: risperidone 6 mg qd; olanzapine 20 mg qd; zuclopenthixol acetate 50 mg/mL, 1 mL/72 h; zuclopenthixol decanoate 200 mg/mL, 1 mL/2 wks

NAD, no abnormality detected; N/D, no data; yo, years old; mo, months old; PANS, positive and negative symptoms scale; qd, once daily; wks, weeks; MRI, magnetic resonance imaging; FL, frontal lobe; OT, occipitotemporal; MRI, magnetic resonance imaging; CT, computed tomography; MMSE, mini-mental state examination; MOAS, modified overt aggression scale; N/D, no data; NAD, no abnormality detected; FAB, frontal assessment battery; TBI, traumatic brain injury; WAIS, Wechsler adult intelligence scale.

## Data Availability

The data presented in this study are available on reasonable request from the corresponding author. The data are not publicly available due to ethical and institutional reasons.
